# Modeling Diabetic Corneal Neuropathy in a 3D *In Vitro* Cornea System

**DOI:** 10.1038/s41598-018-35917-z

**Published:** 2018-11-23

**Authors:** Phillip M. Deardorff, Tina B. McKay, Siran Wang, Chiara E. Ghezzi, Dana M. Cairns, Rosalyn D. Abbott, James L. Funderburgh, Kenneth R. Kenyon, David L. Kaplan

**Affiliations:** 1 Department of Biomedical Engineering, Tufts University, Medford, MA 02155 USA; 2 Department of Ophthalmology, University of Pittsburgh, Pittsburgh, PA 15213 USA; 3 Department of Ophthalmology, Tufts New England Medical Center, Boston, MA USA

## Abstract

Diabetes mellitus is a disease caused by innate or acquired insulin deficiency, resulting in altered glucose metabolism and high blood glucose levels. Chronic hyperglycemia is linked to development of several ocular pathologies affecting the anterior segment, including diabetic corneal neuropathy and keratopathy, neovascular glaucoma, edema, and cataracts leading to significant visual defects. Due to increasing disease prevalence, related medical care costs, and visual impairment resulting from diabetes, a need has arisen to devise alternative systems to study molecular mechanisms involved in disease onset and progression. In our current study, we applied a novel 3D *in vitro* model of the human cornea comprising of epithelial, stromal, and neuronal components cultured in silk scaffolds to study the pathological effects of hyperglycemia on development of diabetic corneal neuropathy. Specifically, exposure to sustained levels of high glucose, ranging from 35 mM to 45 mM, were applied to determine concentration-dependent effects on nerve morphology, length and density of axons, and expression of metabolic enzymes involved in glucose metabolism. By comparing these metrics to *in vivo* studies, we have developed a functional 3D *in vitro* model for diabetic corneal neuropathy as a means to investigate corneal pathophysiology resulting from prolonged exposure to hyperglycemia.

## Introduction

Diabetes mellitus is characterized as a group of metabolic diseases associated with the body’s inability to produce or react to insulin, leading to prolonged high blood glucose. An estimated 30.2 million Americans are diagnosed with diabetes with growing worldwide prevalence and over 1.5 million new cases reported every year^1^. Diabetes development is associated with a number of comorbidities, including cardiovascular disease, stroke, and kidney disease, which remain major contributors to the death toll in developed countries^1^. Likewise, unmanageable eye diseases, including diabetic retinopathy, cataracts, and macular edema, also develop as a result of chronic hyperglycemia in 28–60%^2,3^ of Type 2 diabetic patients, which can lead to visual impairment or even blindness. Corneal defects often develop concurrently with generalized diabetic polyneuropathy, initially presenting as neurotrophic keratopathy and eventually causing to profound degeneration of corneal innervation^4,5^. The morphological changes in sensory nerves associated with prolonged hyperglycemia have been well-characterized in various tissues with loss in nerve endings and increased tortuosity of the remaining fiber bundles^6^. Within the cornea, sensory neurons directly influence the integrity of the corneal epithelium, stroma, and endothelium, slowing or halting mitosis if damaged and leading to reduced tissue regeneration^7,8^. Eventually, the presence of peripheral nerve damage can give rise to epithelial degeneration as the epithelial cells swell, lose microvilli, and produce abnormal basal lamina resulting in recurrent corneal erosions, keratitis, and persistent epithelial defects due to decreased corneal sensation^9^. Suppressed wound healing and re-epithelialization commonly associated with diabetes are assumed to be due to the presence of abnormal adhesions between the epithelium and the underlying basement membrane, creating a greater risk of developing neurotrophic corneal ulceration^10^.

Mechanistically, the polyol pathway has been linked to diabetic complications in the neural retina and lens through the production of excess reactive oxygen species (ROS) and reduced glutathione availability, causing osmotic damage^11^. Development of diabetic neuropathy has also been linked to increased activation of protein kinase C (PKC) by means of the diacylglycerol (DAG)-PKC pathway. During hyperglycemia, an increase in glycolysis leads to increases in *de novo* DAG synthesis, enhancing PKC activation. Elevated PKC levels influence several important physiological processes, including release of transcription factors involved in fibroblast cell migration, growth, proliferation, as well as extracellular matrix (ECM) remodeling^12,13^. Upregulation in the expression of PKC isoforms contributes to the pathogenesis and progression of diabetic neuropathy, as well as other pathways involved in inflammation, fibrosis and hypertrophy^13,14^. Increased pro-inflammatory factors, interleukin-1β (IL-1β) and tumor necrosis factor-α (TNF-α), exacerbate tissue damage during diabetes via the recruitment of leukocytes to affected tissues^15,16^. Studies of corneal fibroblasts *in vitro* suggest that IL-1β stimulation may drive stromal ulceration via activation of matrix metalloproteinases (MMPs)^17^. Furthermore, inhibition of IL-1 receptor in diabetic mice has been associated with reduced complications, suggesting a functional role for this pro-inflammatory factor in diabetes-associated complications^18^.

While *in vivo* studies can provide the most accurate physiological details of diabetic neuropathy, the system complexity may be detrimental to studying transitory molecular events involved in cell death or survival under glucose stress^19^. Hence, the use of more controlled *in vitro* systems potentially circumvents these problems, though very few models have been explored thus far^19^. Given disease pervasiveness and a dearth of appropriate systems, though, a clinical need has arisen to establish accurate tissue models for diabetic research. Some such approaches employing tissue engineered *in vitro* corneal models include a corneal equivalent for drug permeation studies^20^, and an innervated model to examine nerve-target cell interactions^21^. Also, the most extensively used methodology for *in vitro* models of general neuropathy is a primary culture of dorsal root ganglion (DRG) and the neuroblastoma cell line^19,22–25^. Prior *in vitro* diabetic studies using primary cultures of DRG neurons required 25 mM D-glucose in medium for optimal survival, and needed at least 45 mM glucose to induce apoptosis^22,26,27^. Thus, high glucose treatment medium consists of an additional 20 mM D-glucose supplemented to standard medium concentrations. Physiologically, the 45 mM glucose treatment is in fact a comparable fold change relative to the blood glucose concentration difference in a person diagnosed as diabetic (an increase of 1.8 versus ≥1.4 above controls respectively)^22^. An equivalent glucose concentration was then evaluated for human sensory neurons in this study to maintain consistency with other models of hyperglycemia and human diabetes. The tissue model system utilized may influence sensitivity to hyperglycemia with toxicity dependent on cell type, exposure time (acute versus chronic), and culture conditions (2D monocultures versus 3D tissue models versus animal models).

**Figure 1:**
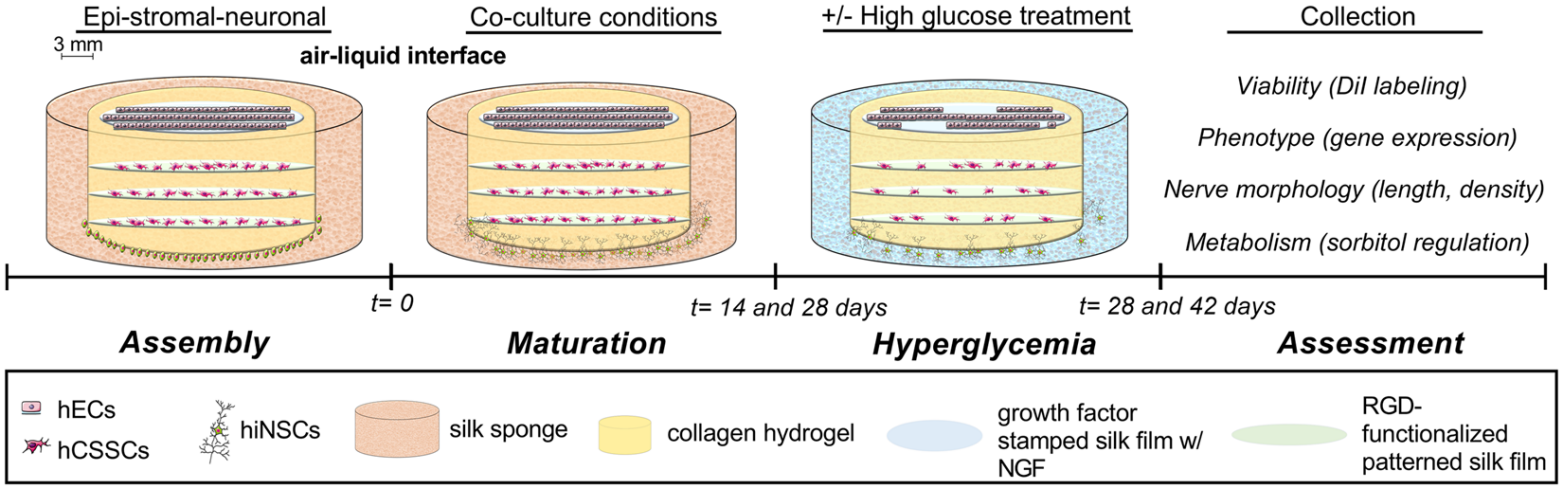
Experimental design to assess the effects of acute hyperglycemia on corneal tissue structure *in vitro*. Extension of nerve fibers are evident by 14 days post-construct assembly with growth continuing up to 42 days. Effects of high-glucose conditions were evaluated to determine if elevated environmental glucose levels (shown in blue) mediate changes in cell viability, phenotypic markers, and nerve morphology. Cell structures generated using Servier Medical Art licensed under a Creative Commons Attribution 3.0 Unported License (https://creativecommons.org/licenses/by/3.0/).

Our lab previously developed a novel innervated 3D corneal tissue model that supported sustained co-culture of corneal epithelial and stromal layers *in vitro* with dense innervation into the upper epithelium (Fig. 1)^28^. Recent characterization of this corneal tissue model has been reported by our lab^29^ showing validation of a functional response with secretion of Substance P following stimulation with established pain activators, such as capsaicin and low-dose sodium lauryl sulfate. Furthermore, we have validated that the neurons utilized in our study express sensory neuronal markers, including calcitonin gene-related peptide receptor (CRCP), brain-derived neurotrophic factor (BDNF), transient receptor potential cation channel subfamily V member 1 (TRPV1), and neurotrophic receptor tyrosine kinase 1 (NTRK1)^29^, thereby supporting the functionality of our model in the study of nociception and pain assessments. In our current study, we investigated the morphological and functional effects of prolonged hyperglycemia on nerve degeneration within the cornea. By varying the culture time and glucose concentration, nerve morphology was compared in hyperglycemic and euglycemic environments. We further characterized epithelial and stromal survival, phenotypic marker expression, neuronal dendrite density and morphology, and expression of select metabolic enzymes associated with the polyol pathway and PKC. Downstream effects of the diabetic disease state on the tissue models were quantified using the pro-inflammatory markers, IL-1β, TNF-α, and MMP-9. The results of our study demonstrate a significant improvement upon past methods for visualizing diabetic neuropathy *in vitro* and present a novel approach to study diabetes-associated complications within the peripheral nervous system. Moreover, development of this *in vitro* tissue model to mimic the effects of hyperglycemia on corneal innervation suggests further potential application of this bioengineered system for broader studies of chronic nerve function and dysfunction.

## Results

### Effects of hyperglycemia on epithelial and stromal layers

**Figure 2:**
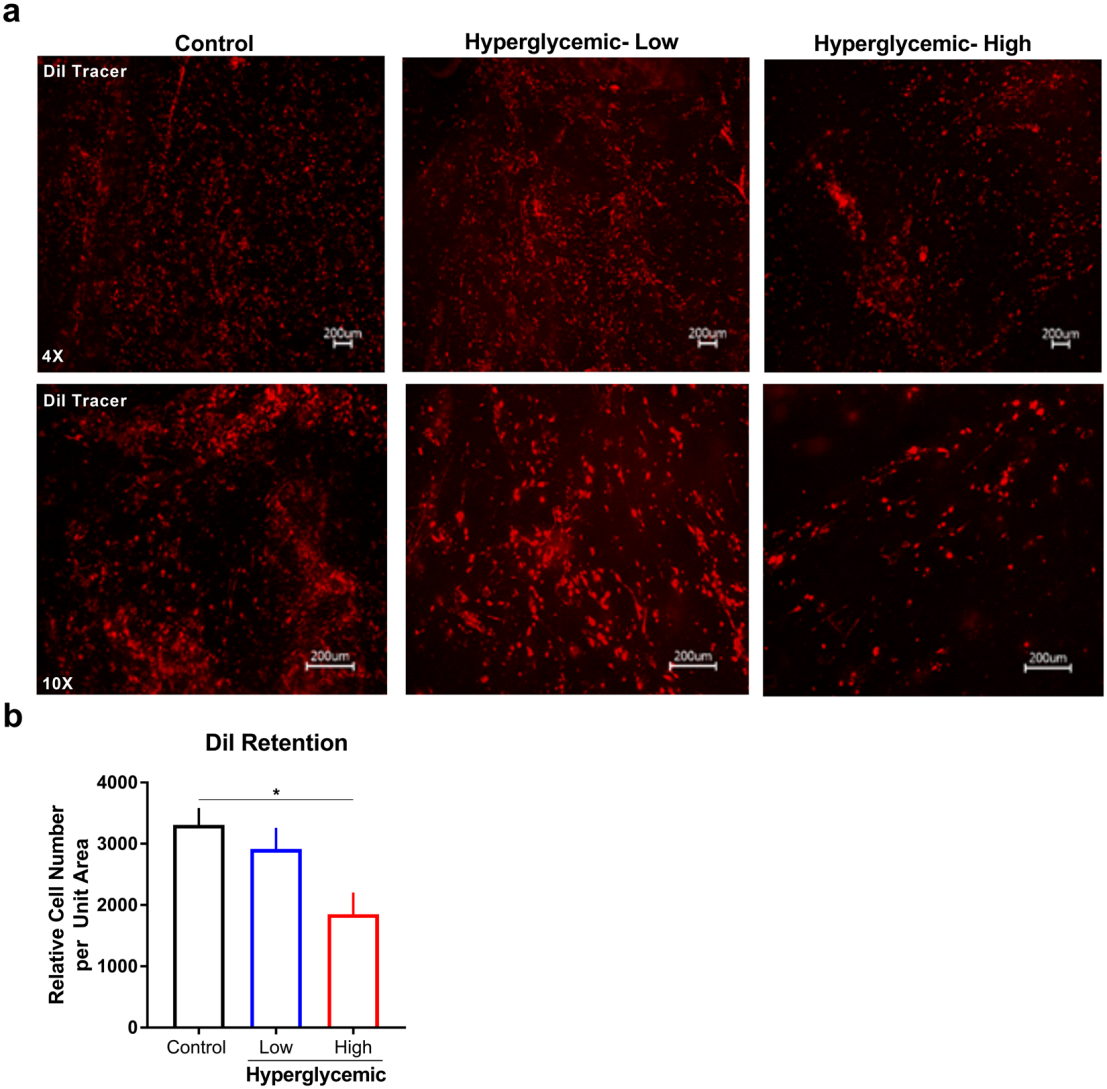
Relative epithelial and stromal cell number in control and hyperglycemic conditions at day 28 in co-culture. Hyperglycemic conditions defined as low (35 mM) and high (45 mM) glucose exposure compared to euglycemic media. (**a**) Fluorescent labeling using Vybrant™ DiI Cell-Labeling Solution (red) as a measure of cell survival and proliferation. (**b**) Quantification of relative cell number determined using particle analysis in ImageJ (n = 3). Error bars represent standard error. *p < 0.05 as determined by a one-way ANOVA.

To evaluate the epithelial and stromal survival *in vitro*, cells were labeled using Vybrant™ DiI Cell-Labeling fluorescent dye prior to seeding on silk substrates as a means to visualize cell retention and proliferation following long-term culture conditions. After stimulating corneal constructs for 14 days with hyperglycemic media in a 28 day total co-culture period, the live cultures were imaged to determine the effects on cell number (Fig. 2a). We found that hECs and hCSSCs in control constructs appeared dense and attached on silk films, having typical shape exemplified by extended, polygonal hCECs and elongated structure for hCSSCs. Similar to studies reporting *in vivo* diabetic changes^10^, high glucose conditions (45 mM) showed a significant reduction in epithelial and stromal cell density by 1.8-fold (p ≤ 0.05), with no significant difference detected with lower hyperglycemic exposure (35 mM) (Fig. 2b). Diffusion of the tracing dye over 28 days may influence detected levels given effects of cell proliferation on loss of the signal in addition to cell death. Further experiments are required to distinguish the select susceptibility of the epithelium and stromal layers to hyperglycemia-induced cell loss.

**Figure 3:**
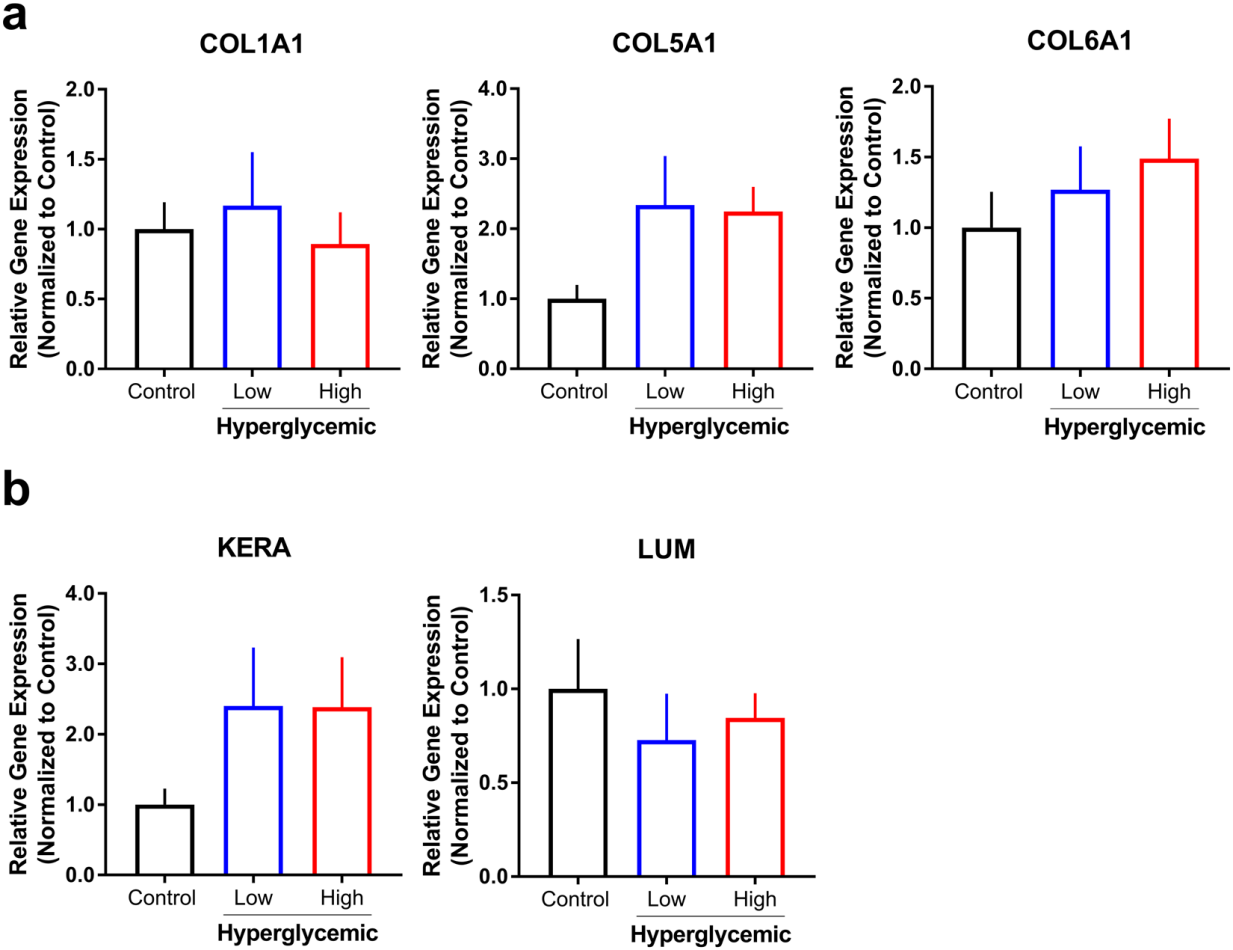
Effects of hyperglycemia on gene and protein expression at day 42 following co-culture. Low (35 mM) and high (45 mM) glucose conditions were evaluated. Relative gene expression of (**a**) collagens (COL1A1), V (COL5A1), and VI (COL6A1), and (**b**) proteoglycans keratocan (KERA) and lumican (LUM) by stromal hCSSCs was determined using the ddCt method normalized to control (standard euglycemic media) with18S used as the housekeeping gene. n = 3–5.

Since the predominant structural components within the human cornea consist of collagens and proteoglycans secreted and assembled within the stroma, we sought to investigate the downstream effects of hyperglycemic conditions on ECM gene expression in our co-culture system (Fig. 3). A key advantage of our model includes defined regions containing specific cell populations based on bioengineered scaffolds, *i*.*e*. neurons localized to the peripheral silk sponge, stromal keratocytes seeded on porous silk films, and the corneal epithelium present on the anterior silk film. Distinct cell types, including the epithelium, hCSSCs, and sensory nerves, were isolated and analyzed separately to account for changes in expression over 4 weeks, normalized to 18S, and compared to controls. For all groups, samples were collected at day 42 after 14 days of high glucose treatment. To evaluate possible “browning” and the production of abnormal basal lamina and ECM composition, the dominant collagen types present in the cornea (collagen I and V)^30^ and collagen VI were analyzed for mRNA expression. Types I, V and VI collagen (COL1A1, COL5A1 and COL6A1) were measured within the stromal section, revealing no significant changes due to glucose stress. Though not significant, COL5A1 was substantially increased for both 35 mM and 45 mM treatment groups (~2.34 and 2.24-fold change respectively). COL1A1 remained largely unchanged in the stroma, and COL6A1 demonstrated only slight trends of increased COL6A1 expression, suggesting hyperglycemic stress may contribute to limited outcomes on stromal ECM expression by hCSSCs *in vitro* (Fig. 3a).

Corneal stromal proteoglycans, keratocan (KERA) and lumican (LUM) play a functional role in regulating collagen fibril assembly in the stroma^31^. To study the effects of high glucose conditions on phenotypic marker expression of hCSSCs, mRNA levels of keratocan and lumican proteoglycans were assessed (Fig. 3b). Under hyperglycemic conditions, keratocan expression was slightly upregulated for treatment groups compared to control with no change in lumican expression. Given previous reports suggesting hyperglycemia increases MMP activity *in vitro*^32^, we sought to determine whether MMP-9 protein levels were altered. We identified trends of lower MMP-9 in low (35 mM) and high (45 mM) glucose conditions suggesting that hyperglycemia may modulate activity independent^32^ of expression directly (Supplemental Fig. 4). Diabetes has been associated with elevated inflammation^33^, thus, we also explored the effects of high glucose on pro-inflammatory factor expression in our model. A significant upregulation in IL-1β (p ≤ 0.01) expression was identified with high hyperglycemia (45 mM) independent of a change in TNF-α expression (Supplemental Fig. 4). The epithelium is the dominant producer of IL-1β in the cornea following wounding or infection^34^ agreeing with our data showing no detectable levels of IL-1β under normal glucose conditions. Studies showing a role for IL-1β and the receptor IL-1Ra suggest that this biochemical pathway may play an important role during wound healing in the diabetic cornea^35^. Our results suggest that hyperglycemia-induced upregulation of IL-1β independent of resident immune cells may promote a pro-inflammatory microenvironment *in vitro*. Further characterization of the inflammatory response to hyperglycemia, as well as nerve degeneration, is required to validate the role of other key cytokines important in corneal homeostasis, including IL-1α and IL-6.

### Hyperglycemia induces altered nerve density and morphology

**Figure 4:**
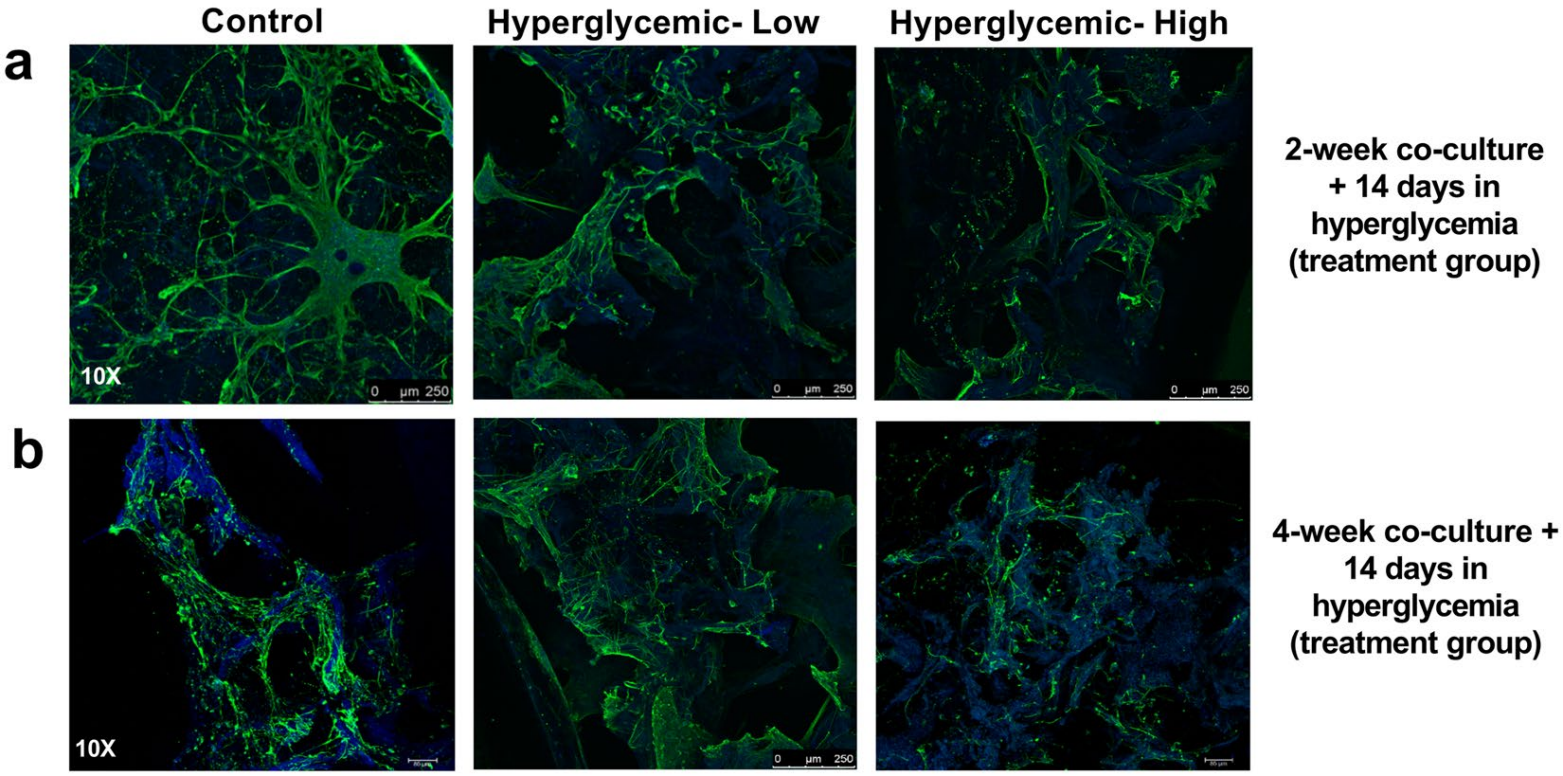
Morphological differences in neuronal extensions following exposure to hyperglycemic conditions. Representative images of immunohistochemistry staining of β III tubulin (green) and DAPI (blue) demonstrating the differences in axon length and cell density between hyperglycemic (low-35 mM and high-45 mM glucose) and control group. Silk autofluorescence is apparent in all images as shown in blue.

To determine the effects of hyperglycemia on nerve structure and density as a measure of corneal neuropathy, we probed for β III tubulin expression, a key neuronal marker, and counterstained with DAPI (Fig. 4). Using varying construct maturity, we assessed the effects of hyperglycemia at days 14, 28 and 42 to determine concentration and culture-time dependent effects on diabetic-related damage. Samples were cultured on two timelines for comparison, a 28 day group and a 42 day group (to determine if a more mature tissue was required to evaluate disease conditions), and the final 14 days both groups were subjected to hyperglycemia (low-35 mM, high-45 mM glucose-supplemented media) before imaging. Innervation was developed in most co-culture samples, though visible nerve fiber degradation occurred in the 45 mM group. Nerves were visually less circuitous and contracted, having fewer extensions than control groups for both timelines. Tissues under high glucose concentrations also had nerve loss, leaving sparse regions within the sponge. As shown, control and 35 mM axon lengths were long and tortuous within sponges, and in some regions guided toward innervating the inner epithelial layers (Fig. 4a,b). However, in the 45 mM group, axon lengths were absent, with neurons remaining in the sponge instead of innervating inner epithelium (Fig. 4c).

**Figure 5:**
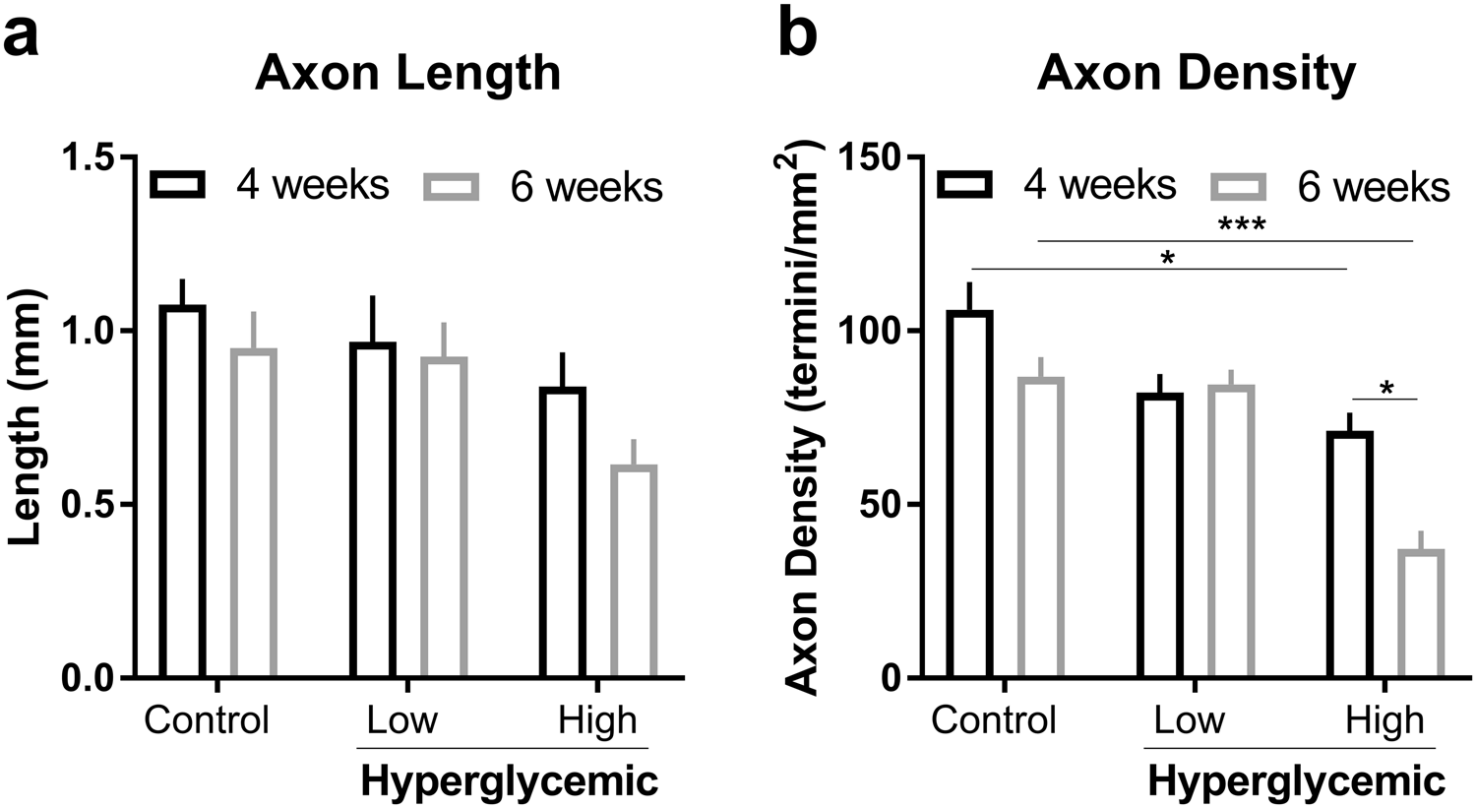
Quantification of length (**a**) and density (**b**) of axons in day 28 and day 42 co-culture under high glucose concentrations for 14 days using 10X confocal images. Data was collected from n = 3 samples per condition and 5 regions per scaffold. Measurements and densities were determined using the ImageJ software plugin NeuronJ. Standard deviation is indicated as error bars for each group. *p < 0.05, **p < 0.005 and ***p < 5E-6, one-way ANOVA with a *post-hoc* Tukey test.

To determine the extent to which innervation is affected by diabetic conditions, scaffolds were imaged using confocal microscopy to measure axon length and density with different treatments (Fig. 5 and Supplemental Fig. 3). The axon density and lengths were calculated for both experimental timelines to compare the impact of high glucose on nerve fiber damage. Particularly, at day 42, high glucose-treated samples reached on average 0.616 ± 0.124 mm and 37.2 ± 9.1 termini/mm^2^ versus 0.95 ± 0.182 mm and 86.8 ± 9.7 termini/mm^2^ for the control group as measured by 10X confocal images. Consequently, axon lengths were significantly lower in the high glucose group (0.616 ± 0.124 mm) compared to the control group (0.95 ± 0.182 mm) (one-way ANOVA). There was no statistically significant difference between the day 28 axon lengths suggesting that longer culture times may influence susceptibility of sensory neurons to hyperglycemic-induced damage. Similar analysis was also applied for nerve density, demonstrating a statistically significant difference between groups 45 mM groups (71.2 ± 9 and 37.2 ± 9.1 for day 28 and day 42, respectively) and controls (106 ± 14 and 86.8 ± 9.7 for day 28 and day 42, respectively) (one-way ANOVA) at both time-points thereby recapitulating hyperglycemic-induced nerve degeneration *in vitro*. At higher levels of glucose (55 mM), we found an absence of neuronal extensions with diminutive signs of neuron growth, as most sponges were acellular suggesting that exposure to excess glucose at these levels *in vitro* may be too severe for representing chronic effects of hyperglycemia (Supplemental Fig. 1).

### Differentiated hiNSC neurons as a primary target for hyperglycemia

**Figure 6:**
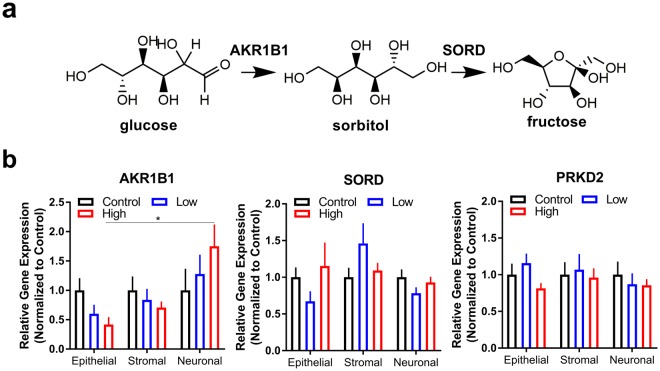
Perturbation of glucose metabolism in neurons during diabetes favors an upregulation in aldose reductase, the enzyme responsible for sorbitol production. (**a**) Schematic of sorbitol and fructose biosynthesis during excess glucose conditions. (**b**) Gene expression of key metabolic enzymes in day 42 co-culture: aldose reductase (AKR1B1) and sorbitol dehydrogenase (SORD). Protein kinase D2 (PRKD2) expression as a key regulator of proliferation. n = 3, *p < 0.05 as determined by a one-way ANOVA.

Select metabolic enzymes in the polyol pathway have been associated with diabetes-related complications due to upregulation in sorbitol and depletion of reduced glutathione levels^36^. We assessed changes in gene expression of sorbitol dehydrogenase (SORD) and aldose reductase (AKR1B1) as an indirect measure of polyol pathway flux and, along with protein kinase C (PKD2), as key characteristic markers for diabetic neuropathy (Fig. 6a). For epithelial and stromal layers, the fold change in expression of SORD and PKD2 was unchanged, and AKR1B1 was similarly expressed compared to control (Fig. 6b). Interestingly, neuronal sections showed a significant upregulation in aldose reductase in high hyperglycemic conditions compared to the epithelial layer. Thus, sensory nerves may have increased susceptibility to the diabetic microenvironment compared to the epithelium and stroma. With no significant change in sorbitol dehydrogenase or protein kinase C measured in any cell type, our results support previous literature positing that an accumulation of sorbitol may be a primary constituent of diabetic ocular complications within the anterior segment^37^. Further studies are required to determine mechanistic details regarding the role of AKR1B1 in nerve degeneration.

## Discussion

We report having developed a functional model to mimic diabetic corneal neuropathy *in vitro* using silk scaffolds as a mechanically tunable material comparable to collagen-based approaches and corneal elasticity found within the human cornea^21,38^. Utilizing this approach, we examined degeneration of nerve fibers that arise in the corneal constructs following prolonged hyperglycemia and found a significant reduction in neuronal outgrowth and extension in high glucose conditions. Of importance, longer term 42 day culturing timelines were supported in our system, providing evidence that the novel corneal constructs remained viable during sustained cultivation and thereby permitting chronic studies^28^. Control hCSSCs maintained alignment and density throughout the experimental timeline (≤6 weeks, data not shown) with hyperglycemia inducing altered keratocyte survival. Regions of the 35 mM scaffolds were unchanged in cell density after 28 days, with significant changes in cell count found throughout 45 mM samples. Results demonstrated successful differentiation of hCSSCs into keratocytes with KERA and LUM genes being highly expressed and only modest effects of hyperglycemia on collagen or proteoglycan expression. Therefore, our tissue model suggests that ECM assembly by stromal keratocytes may not be directly regulated by glucose conditions utilized in our system. Our results suggest that high glucose alone does not promote changes in expression of these genes, thus supporting the hypothesis that chronic changes that occur in the corneal microenvironment, such as nerve degeneration, may promote these changes independent of extracellular glucose levels. Further studies are required to validate that longer exposure to hyperglycemia (on the scale of months to years) does not alter the keratocyte phenotype and stromal matrix independent of nerve loss. As previously reported in both *in vivo* human and animal models for corneal neuropathy, diabetes is associated with epithelial thinning and, due to edema, increased in stromal thickness^20,21^. To recapitulate this phenomenon *in vitro*, the addition of an endothelial layer may be warranted to study how hyperglycemia affects water retention within the cornea.

As determined by fluorescence microscopy staining for β III tubulin, hiNSC neurons sufficiently grew into the inner layers of the scaffolds, confirming innervation within the model. After 28 days, control groups demonstrated axonal branching, elongation, and sprouting, and were on average 1.08 mm in length having 106 termini/mm^2^. Amongst the 45 mM treatment group, axons were significantly smaller at day 42 and less dense under both timelines, implying nerve fiber impairment. Morphologically, nerve tortuosity was visibly reduced, and neuronal degradation was consistent with the damage and sparse regions of nerve fiber loss reported in 18-week STZ injected mice^21^. This reduction in both nerve and fiber density in high glucose conditions correlates with clinical features associated with diabetic neuropathy. Loss in neurotrophic input has consequential effects on corneal tissue regeneration via regulation of limbal stem cell proliferation of the epithelium. Further characterization of our tissue model may serve to define the role of sensory innervation during physiological corneal tissue maintenance and pathological processes, such as dry eye disease, herpes simplex infection, and LASIK-associated keratitis.

Since diabetes is a metabolically-driven disease, downstream effectors of excess glucose play an important role in defining the means by which a cell processes excess glucose. In our model, we found hyperglycemia increased the polyol shunt by a 1.75-fold change in gene expression for AKR1B1 in neurons at day 42, which is analogous to fold changes from previous long term animal and human studies^39,40^. Given the dependence of AKR1B1 on NADPH as a co-factor, excessive elevation in sorbitol levels may be an indirect means for glutathione depletion, the major antioxidant present to re-generate NADPH from NADP+. SORD expression remained unchanged correlating with previous studies showing sorbitol accumulation during diabetic complications^19,41^. Therefore, the early stages of hyperglycemia do not necessitate an increase in sorbitol dehydrogenase levels. Gene expression of PKD2 remained statistically unaffected at day 42 for all scaffold regions, suggesting the DAG-PKC pathway was not changed upstream. Under glucose stress *in vivo*, PKC is activated by DAG synthesis due to an increase in glycolysis flux. Activation of PKC is not only purported to influence key processes involving proliferation and ECM deposition, but angiogenic factors as well. These include the release of vascular endothelial growth factor (VEGF), vascular permeability, endothelial cell growth, and leukocyte stimulation and adhesion (leukostasis) contributing in neovascularization and irregular blood flow^12–14^. While we observed no change in PKD2 expression in our model, this may have resulted from the absence of an endothelial layer or temporal effects of hyperglycemic-induced PKC activation.

Multiple reports have investigated the mechanisms leading to diabetic neuropathy using DRGs cultured in collagen-coated plates^22^ with studies of the effects of diabetes on the corneal stroma investigated in a self-assembled *in vitro* model^42^ and *ex vivo* approaches using human corneal buttons isolated from diabetic patients^43,44^. As exemplified by transcript and phenotypic changes, the additional complexity of our current co-culture system more accurately mimics innervation present *in vivo* in a 3D *in vitro* model. In comparison to former *in vitro* approaches, past research has identified many biochemical markers emblematic of diabetic neuropathy that have yet to be quantified within the *in vitro* corneal tissues, including fluorimetric analysis of ROS production, assays of antioxidant enzymes, and immunocytochemistry to evaluate cell death demonstrating appropriate hyperglycemic conditions^22^. A number of *in vitro* diabetic studies utilize primary cultures of neurons stimulated with >45 mM glucose to prompt apoptosis^22,26,27^, which is congruous with our current study utilizing 45 mM glucose treatment as inducing diabetes-related damage. The 1.8-fold increase over standard glucose medium corresponds to the ~2-fold increase in diabetic patients’ blood glucose^45^. Though immunohistochemistry suggests apoptosis within the cornea co-culture model, further analysis, such as cleaved caspase-3 quantification, is required to explicitly evaluate the disease state.

In using the current *in vitro* corneal model, there are foreseeable limitations, including the prohibitive culture times required for neuronal outgrowth and innervation into the epithelium and stromal layers. In the tissue-engineered cornea, axons developed from the bottom of silk sponge and grew towards the top of the scaffold. This cultivation time was limited to 2–4 weeks before high glucose medium treatment, while embryonic corneal maturity *in vivo* lasts 2 months^46^. Obtaining adequate innervation with sensory neurons is essential and may require longer culture times (>8 weeks) prior to hyperglycemic stress, particularly in circumstances of keratopathy because the deficiency in cellular growth may preclude interactions with the epithelium. Furthermore, changes in the corneal epithelial, stromal, and neuronal cell populations during diabetes occurs during chronic exposure to high glucose with significant fluctuations in hormone levels, such as insulin, that influence the global response to elevated glucose levels^47^. These factors must be considered in the application of *in vitro* systems in the study of diabetic-related complications.

The novelty of our approach has led to the development of a benchtop, tunable *in vitro* tissue model to describe diabetic neuropathy and cellular dysfunction that occurs during hyperglycemia by utilizing a 3D co-culture scaffold constructed from silk materials. The *in vitro* model supported viable innervation and demonstrated clinical relevance by using only human cell types derived from primary sources with functionalization of a silk scaffold to enable cell attachment and alignment, while maintaining stromal transparency. Prior literature reveals a paucity of *in vitro* corneal models simulating diabetic disease states, and no such approach has utilized a tissue-engineered system with all three relevant corneal cell types (epithelial, stromal, and neuronal) that can be maintained in long-term conditions. Corneal tissue innervation was paramount in this research, and silk substrates remained robust throughout the long culture timelines retaining integrity and transparency after 42 days. This 3D *in vitro* platform may serve as a high-throughput approach for assessing therapeutic safety and effectiveness in the context of the peripheral nervous system and ocular tissue enabling investigation into both acute and chronic effects of diabetes.

## Methods

### Ethical and informed consent

All human cells utilized in these studies were commercial-sourced or isolated in-house from de-identified tissues, therefore excluding this study from human subject research in accordance with the relevant guidelines and regulations.

### Preparation of aqueous silk solution

Silk solution was obtained from *Bombyx mori* silkworm cocoons as previously described^48,49^. Briefly, silk cocoons were supplied by Tajima Shoji Co. (Yokohama, Japan) and boiled for 30 minutes in 0.02 M Na_2_CO_3_ (Sigma-Aldrich, St Louis, MO), washed in deionized water, and dried overnight. Extracted fibroin was solubilized using a 9.3 M LiBr solution for 4 to 6 hours in a 60 °C dry oven and dialyzed against 4 L deionized water over 2 days, changing water frequently. The final solution obtained was an aqueous silk solution (5–8% w/v).

### Stamped and patterned silk film preparation

Porous silk films were prepared using 1% w/v silk solution and 0.05% w/v polyethylene oxide (PEO, MW = 900,000, Sigma-Aldrich) in deionized water cast on 12 mm glass coverslips (Electron Microscopy Science, Hatfield, PA)^28^. Films for epithelial growth were stamped using 12 mm polydimethylsiloxane (PDMS) (Fisher Scientific Co. Fair Lawn, NJ) molds dipped in stamping solution composed of 50 μL (4 mg/mL) type I collagen (rat-tail tendon, BD, Franklin Lake, NJ), 100 ng/mL keratinocyte growth factor (KGF) (Sigma), 100 ng/mL hepatic growth factor (HGF) (Sigma), 200 ng/mL epithelial growth factor (EGF) (Thermo Fisher, Waltham MA) and NGF (200 ng/mL) (R&D Systems, Minneapolis, MN). Stamped films were placed in a desiccator to water anneal at 25 mmHg for 2 hrs, UV-sterilized, and incubated in 10 µg/mL poly-D-lysine (PDL) overnight at 4 °C. Larger, patterned silk films for keratocyte growth were cast by using 1.6 cm^2^ polydimethylsiloxiane (PDMS) molds with aligned microgrooves at a density of 600 lines/mm followed by water annealing, PEO leaching and UV-sterilization. Patterned films were functionalized with Arginine-Glycine-Aspartic acid-Serine (RGD) peptides (Bachem, Torrance, CA) and used within one week of functionalization.

### Salt-leached silk sponge preparation

Silk sponges were fabricated with a salt leaching method creating 500–600 nm sized pores using 6% w/v silk solution, dried for 48 hr, heated at 60 °C for 1–2 hr, and dialyzed against 4 L deionized water for 2 days. Sponges were sectioned to 1 mm thickness using a microtome blade a 12 mm and 15 mm biopsy punch (McMaster-Carr, Robbinsville, NJ) to achieve the annulus inner and outer diameter, respectively. Completed silk sponges were autoclaved and soaked in PDL at 4 °C overnight.

### Human corneal stromal stem cell (hCSSC) culture

hCSSCs were isolated from the stromal tissue of human corneas obtained from the Center for Organ Recovery and Education (CORE; Pittsburgh, PA) using collagenase digests, as previously reported^50^. A cell suspension (15,000 cells/cm^2^, passage 4–5) was added to films and incubated for 4 hr to allow for cell attachment. Seeded films were cultured in proliferation media composed of a 3 to 2 ratio of DMEM to MCDB-201 (v/v) with 2% fetal bovine serum (FBS), 10 ng/mL platelet-derived growth factor, 1 mg/mL lipid-rich bovine serum albumin (Albumax, Life Technologies, Grand Island, NY), 10 ng/mL epidermal growth factor, 5 mg/mL transferrin, 5 ng/mL selenous acid, 0.1 mM ascorbic acid-2-phosphate, 10^−8^ M dexamethasone, 100 IU/mL penicillin, 100 μg/mL streptomycin, 50 μg/mL gentamicin, and 100 ng/mL cholera toxin overnight before moving to co-culture.

### Human corneal epithelial cell (hCEC) culture

Primary hCECs purchased from a commercial-source (C0185C, Thermo Fisher, passage 3) were seeded onto the films at a density of 150,000 cells/cm^2^. The films were then incubated for 8 hr to allow cell attachment before culturing in keratinocyte SFM media (Thermo Fisher) 2 days prior to co-culture to reach necessary confluency.

### Differentiated hiNSC Neuron culture

Silk sponges were seeded with human induced neural stem cells (hiNSCs) which were derived by direct reprogramming of dermal fibroblasts as previously described^51^. hiNSCs were generated in-house from de-identified human neonatal foreskin fibroblasts (HFFs) (a gift from Dr. Jonathan Garlick, Tufts University). hiNSCs were propagated on mitomycin C-activated mouse embryonic feeder (MEF) layers in Knockout (KO) DMEM supplemented with 20% KO xeno-free serum replacement, 20 ng/mL recombinant bFGF, 1% Glutamax, 1% antibiotic- antimycotic, and 0.1 mM β-mercaptoethanol. Once confluent, cells were trypsinized using 0.25% trypLE select (Thermo Fisher) from gelatin coated 35 mm dishes. Cells were resuspended to obtain a density of 1–5 million cells per scaffold and added to an acetic acid-type I collagen hydrogel (100 μL of collagen hydrogel and 30 μL of cell suspension was used to seed sponges) and cultured in neurobasal media with 1% glutamax, 2% B-27 and 1% Anti-Anti (Thermo Fisher). Once seeded, cells were differentiated into neurons for 10 days with 3 growth factors (NGF, BDNF, and GDNF) and at 5 days, 3 inhibitors (SU5402, DAPT and CHIR99021)^51,52^. After 10 days of growth, silk seeded sponges were inverted to promote innervation from the bottom of the scaffold. Before co-culture, hiNSCs were moved to differentiation media containing advanced DMEM (Life Technologies), 25 ng/mL NGF (R&D Systems) 1.0 mM l-ascorbic acid-2-phosphate (Sigma), 50 μg/mL gentamicin (Life Technologies), 2 mM L-alanyl-l-glutamine (Life Technologies), 100 μg/mL penicillin, 100 μg/mL streptomycin (Mediatech, Manassas, VA), 0.1 ng/mL transforming growth factor-beta3 (TGFβ-3, Sigma), 10 ng/mL basic fibroblast growth factor (FGF-2, Sigma) and 1% Anti-Anti (Thermo Fisher).

### Co-culture assembly

Co-culture scaffolds were constructed as previously described^28^. Briefly, after the 10 day culture period, silk sponges were inverted and transferred to differentiation media with 50 ng/mL NGF. Three patterned silk films seeded with hCSSCs were stacked on top of one another, which constitute the cornea’s stroma. Collagen hydrogel was employed to cross-link scaffold layers by adding 900 μL (4 mg/mL) acetic acid-type I collagen solution (rat-tail tendon, Corning, Corning NY), 100 μL 10x DMEM (Sigma) and 20 μL 1 M NaOH (Sigma) for neutralization. Alignment of each film layer was such that the patterns were perpendicular to one another, and stacked stromal layers were cut to size using a 12 mm biopsy punch (McMaster-Carr). Stromal film stacks were placed within each of the sponges so that the films were centered within the sponge ring. Stamped epithelial films were then transferred and arranged carefully atop the stroma. Scaffold integrity was achieved by adding 300 μL collagen gel around the circumference, binding the films to the sponge and connecting the model. Finally, to drive neuron innervation upward toward the epithelium, 100 μL collagen hydrogel with 4 μL NGF (750 µg/mL) was pipetted onto the top-most layer. Scaffolds were then incubated for 30 minutes to induce cross-linking before moving the completed model into culture in differentiation media. Custom designed waffle-shaped PDMS inserts (5 mm thick, 5 cm diameter, with 16 × 1 mm^2^ holes) were cast with Delrin molds (McMaster-Carr). Designed for media irrigation and development of an air-liquid interface, a total of three scaffolds were cultured per PDMS insert.

### HT29-MTX culture

For comparing viability in hyperglycemic and euglycemic conditions, the mucosal cell line, HT29-MTX (source: Public Health England Culture Collections (Salisbury, England)) was cultured in DMEM containing 10% FBS and 1X antibiotic/antimycotic for 2 weeks.

### Diabetic model conditions

Scaffolds were exposed to hyperglycemic medium at glucose concentrations similar to previously reported *in vitro* approaches to induce physiologically relevant hyperglycemic damage^53,54^. With the target concentration for diabetic conditions reported as 45 mM, a range of 35 mM, 45 mM and 55 mM glucose supplemented differentiation media was used for the study compared to a low glucose control group cultured in standard 25 mM media^26,53,54^. D-(+)-glucose (≥99.5% GC, Sigma-Aldrich) was added to differentiation media to achieve final working concentrations and filter-sterilized. Scaffolds were maintained for 28 days prior to a 14 day co-culture with hyperglycemic medium. Samples were then collected for analysis to measure markers related to diabetic neuropathy. A shorter 14 day culture timeline before glucose stimulation was also utilized for qualitative measurements by immunofluorescence.

### DiI Staining and Immunohistochemistry

Epithelial and stromal cells were fluorescently labeled using Vybrant® diI cell-labeling (Thermo Fisher) solution before cell seeding for live imaging. Stock diI solution was added at a concentration of 5 μL/mL to cell suspension and incubated for 30 minutes. Imaging scaffolds was performed on a BZX-700 microscope (Keyence Corporation, Itasca, IL) at 10X and 4X magnification. Advanced 3D Analysis software (Keyence Corporation, Itasca, IL) was used to generate maximum intensity projection images. Confocal microscopy was also employed to take images with a Leica SP8 microscope and software suite (Leica Microsystems) at 10X and 20X magnification. Image stacks were merged into single 8-bit tiff files using ImageJ (NIH)^55,56^. Five regions per scaffold were imaged and each sample set was repeated in triplicate. The density of keratocytes was counted using ImageJ cell counter.

For immunohistochemistry scaffolds were fixed in 4% paraformaldehyde (PFA) in PBS (Affymetrix, Cleveland, OH) for 45 min and blocked using 5% BSA and 0.2% Tween-80 in PBS overnight before staining. The following antibodies were incubated with samples overnight at 4 °C: rabbit anti-human βIII-tubulin (1:500, ab52623, Abcam) and goat anti-rabbit IgG Alexa Fluor 594 (1:200, A-11037, Fisher). DAPI was diluted to a concentration of 300 nM in PBS and 0.1% Tween-80 and incubated with samples immediately prior to imaging.

### Neuronal extension measurements

Axon length measurements and densities were determined for 10X images using the algorithm in NeuronJ, an ImageJ software plugin based on previously described methods^57,58^. Briefly, each image was converted to an 8-bit tiff image followed by tracing using the automated function in NeuronJ. Length of axons and densities were measured for each region of interest.

### qRT-PCR

Gene expression levels for keratocan (KERA, Hs00559942_m1), lumican (LUM, Hs00929860_m1), collagen I (COL1A1, Hs00164004_m1), collagen V (COL5A1, Hs00943809_m1) and collagen VI (COL6A1, Hs01095585_m1), sorbitol dehydrogenase (SORD, Hs00162091_m1), aldose reductase (AKR1B1, Hs00739326_m1), protein kinase C (PRKD2, Hs00212828_m1), and the housekeeping gene (18S, Hs03003631, Thermo Fisher) were quantified by RT-PCR. Extracted RNA was reverse transcribed to cDNA using a high-capacity cDNA reverse transcription kit (Thermo Fisher). Quantitative RT-PCR of cDNA (~30 ng/μL) was accomplished with TaqMan fluorescent hybridization probes (Thermo Fisher) for target genes. Relative fold change in gene expression was compared amongst groups using the delta delta cycle threshold (ddCt) method with euglycemic media as a control and 18S as a reference gene.

### Protein Expression

Constructs were isolated and homogenized in M-PER™ Mammalian Protein Extraction Reagent (Thermo Fisher) containing 1X protease inhibitor cocktail (Sigma) for 30 minutes on ice to ensure complete cell lysis. Cell lysates were centrifuged at 12,000 rpm for 15 minutes at 4 °C to pellet insoluble debris, and supernatants were aliquoted and stored at −20 °C until further use. Protein expression was measured using commercial ELISA kits: IL-1 beta Human Uncoated ELISA Kit (Thermo Fisher, 88-7261-22), TNF alpha Human Uncoated ELISA Kit (Thermo Fisher, 88-7346-22), and MMP9 Human ELISA Kit (BMS2016-2) according to the manufacturer’s protocol.

### Viability Assay

Viability of 2D monocultures of hCECs, hCSSCs, and HT29-MTX cultures were assessed using a commercial MTT assay based on the manufacturer’s protocol (Vybrant® MTT cell proliferation assay, Life Technologies, Molecular Probes, Eugene, OR). Briefly, cells were cultured at a density of ~10^4^ cells in each well of a 24-well plate and stimulated with hyperglycemic media (35 mM glucose) for 5 days. Media was then aspirated and samples incubated with the MTT reagent ((3-(4,5-dimethylthiazol-2-yl)-2,5-diphenyltetrazolium bromide) tetrazolium) for 1 hour at 37 °C/5% CO_2_. Following removal of the media, cells were lysed in DMSO, followed by a 20 minute incubation, and absorbance measurements at 540 nm.

### Statistical analysis

A one-way analysis of variance (ANOVA) was applied for all experimental versus control group comparisons, and a *post-hoc* Tukey test was utilized for statistical significance between individual groups using GraphPad Prism 7. The significance level was set at *α* < *0*.*05*. Each of the experiments were completed in at least triplicate (n ≥ 3).

## Electronic supplementary material

Supplemental Data

## Data Availability

All relevant data are included in the manuscript. Materials, data, and protocols described within the paper are available upon reasonable request to the corresponding author.
