# Supplementary material for: Modeling Diabetic Corneal Neuropathy in a 3D In Vitro Cornea System
Source: Sci Rep. 2018 Nov 23;8:17294. doi: 10.1038/s41598-018-35917-z (PMC6251923; doi:10.1038/s41598-018-35917-z)
Supplement: Supplementary file 1 — Supplemental Data [file 41598_2018_35917_MOESM1_ESM.docx]

**Modeling Diabetic Corneal Neuropathy in a 3D *In Vitro* Cornea System**

Phillip M. Deardorff^a^, Tina B. McKay^a^, Siran Wang^a^, Chiara E. Ghezzi^a^, Dana M. Cairns^a^, Rosalyn D. Abbott^a^, James L. Funderburgh^b^, Kenneth R. Kenyon^c^, David L. Kaplan^a,*^

*^a^Department of Biomedical Engineering, Tufts University, Medford, MA 02155, USA*

*^b^Department of Ophthalmology, University of Pittsburgh, Pittsburgh, PA 15213, USA*

*^c^ Department of Ophthalmology, Tufts New England Medical Center, Boston, MA, USA*

^*^Corresponding author: David L. Kaplan, Ph.D., Tufts University, 4 Colby Street, Medford, Massachusetts 02155 USA

**
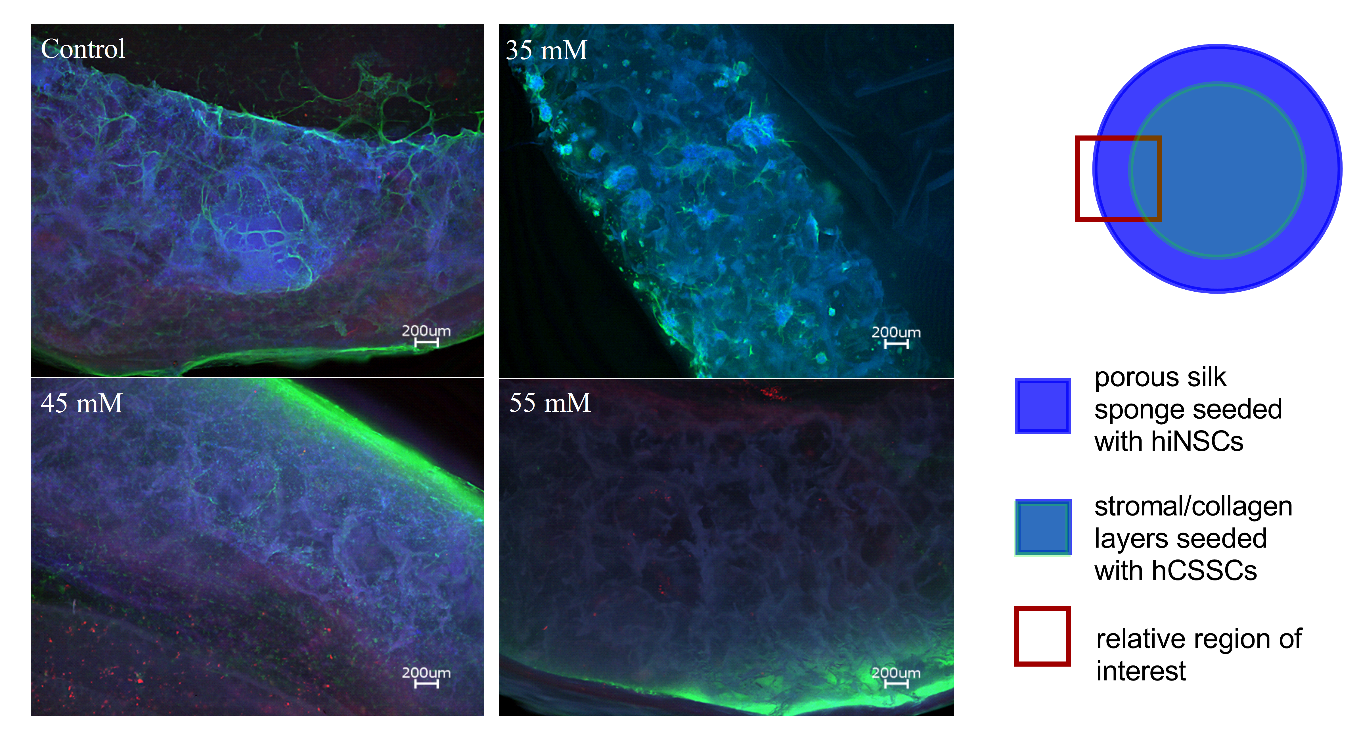
Supplemental Fig 1.** Immunohistochemistry staining of β III tubulin (green) and DAPI (blue) demonstrating the differences in axon lengths and cell density between the various glucose treatment and control groups. Note the lack of neurons present within the sponge for the 55 mM group, suggesting the treatment concentration was too high and caused cell death. Schematic depicts general location of imaging along the sponge-stromal interface. All images shown are under 4X objective lens magnification and scale bars = 200 μm.

**
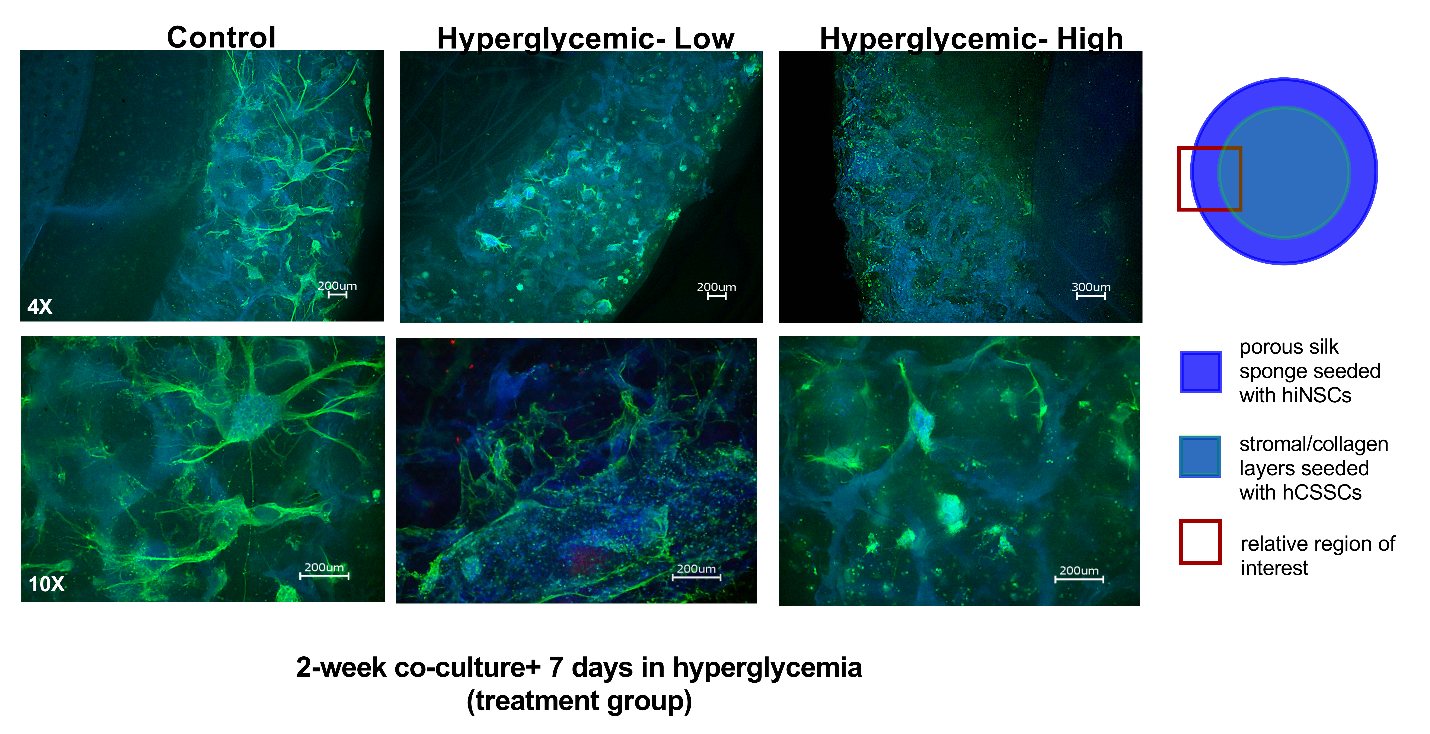
**

**Supplemental Fig 2.** Immunohistochemistry staining of β III tubulin (green) and DAPI (blue). Schematic depicts general location of imaging at the peripheral sponge-stromal interface. Silk autofluorescence is apparent in blue. Magnification of the objective lens (4X and 10X) used during imaging.

**
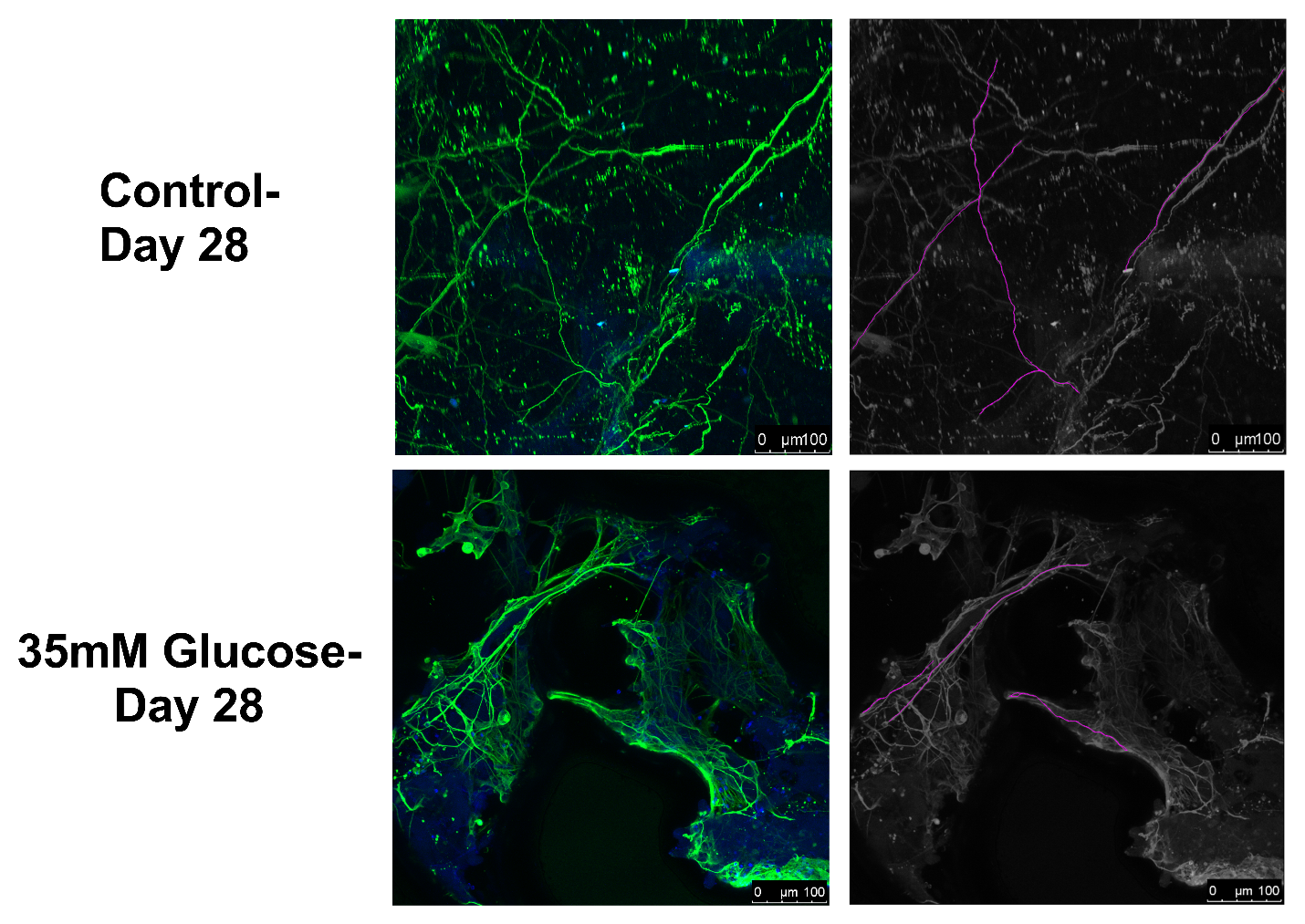
**

**Supplemental Fig. 3.** Representative images of neuronal extension tracings generated using the ImageJ plugin, NeuronJ. Tracings of select neurons shown in purple generated from an 8-bit tiff file.


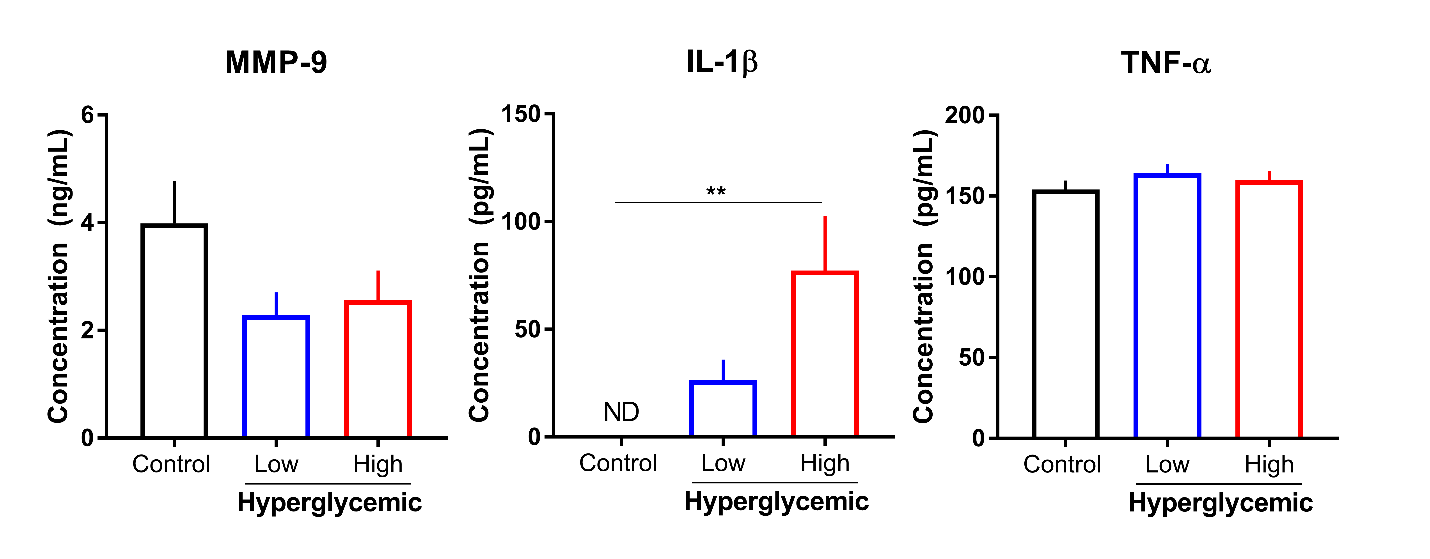


**Supplemental Fig. 4.** Protein expression of matrix metalloproteinase-9 (MMP-9), interleukin-1β (IL-1β), and tumor necrosis factor-α (TNF-α), as determined by ELISA. Corneal constructs were cultured for 3 weeks under euglycemic conditions (25 mM glucose) and transferred to hyperglycemia (35mM and 45mM glucose, low and high, respectively, in treatment groups) for 1 week. n=4-7 based on two-independent experiments. ND= not detected, below the standard curve of detection. Error bars represent standard error. **p < 0.01 as determined by a one-way ANOVA.


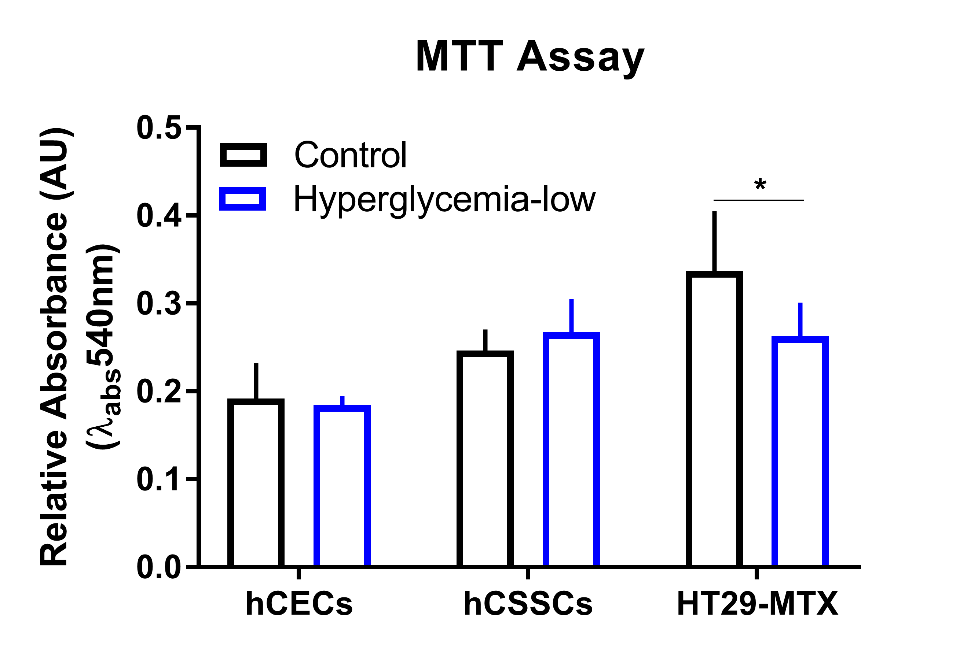


**Supplemental Fig. 5.** Effects of hyperglycemia (35mM glucose) on cell viability post-5 day exposure based on an MTT assay applied to 2D monocultures of human corneal epithelial cells (hCECs), human corneal stromal stem cells (hCSSCs), and a mucosal cell line (HT29-MTX). Statistical significance based on a two-way ANOVA with *p<0.05. Error bars represent standard deviation with n=6.
